# Attachment style and food addiction: Exploring psychological links

**DOI:** 10.1192/j.eurpsy.2025.954

**Published:** 2025-08-26

**Authors:** A. Karmous, H. Ghabi, M. Karoui, H. Nefzi, R. Kammoun, F. Ellouze

**Affiliations:** 1 emergency and outpatient department; 2 Department G, Razi hospital, Manouba, Tunisia

## Abstract

**Introduction:**

Behavioral addiction is well-established for gambling but still debatable for other pleasurable behaviors such as eating. Attachment style is defined as a psychological concept describing the dynamics of human interpersonal interactions. Studies investigating the influence of attachment style on food addiction are rare.

**Objectives:**

The aim of the study was to evaluate the association between food addiction and the quality of attachment

**Methods:**

A cross-sectional study was conducted online with a non-clinical population. All participants completed an anonymous e-questionnaire containing sociodemographic data, background, substance use and self-reported weight and height. Attachment style was assessed with the Relationship Scale Questionnaire (RSQ). Food addiction was screened with modified Yale food addiction scale questionnaire (mYFAS 2.0).

**Results:**

A total of 114 individuals had fully responded to the questionnaire. The mean age was 32.28 ± 9.32 years with a sex ratio of 0.48. The mean BMI was 23.7 kg/m2 ± 5.5. Most of participants (64%) had an insecure attachment style and 36% had a secure one. The results of the mYFAS 2.0 showed that 11.4% of participants had a food addiction and 8.8% had a severe form. BMI was significantly associated with food addiction which was more common in participants who had a BMI greater than 22.2 kg/m2. A statically significant association between insecure attachment style and food addiction was found.

**Conclusions:**

Our study showed the importance of studying attachment style in food addiction. More research is needed to prove the association between food addiction and different attachment styles.

**Disclosure of Interest:**

None Declared

